# Wearable near field communication platform with a battery-free skin-interfaced tag for continuous temperature and impedance monitoring

**DOI:** 10.1557/s43579-026-01029-3

**Published:** 2026-08-20

**Authors:** Chi Han, Shreesha Hanagud, Hunter Zhang, Fuying Dong, Xinnian Jiang, Di Wu, Jason Y. Fang, Simiao Niu

**Affiliations:** 1 Department of Biomedical Engineering, Rutgers University, Piscataway, NJ 08854 USA; 2 Department of Electrical and Computer Engineering, Rutgers University, Piscataway, NJ 08854 USA; 3 Department of Chemical and Materials Engineering, University of California Merced, Merced, CA 95343 USA

**Keywords:** Bioelectronic, Biomedical, Flexible, Sensor, Interface

## Abstract

Battery-free skin-interfaced sensing requires lightweight passive tags that can operate with wearable readers. Here, we report a flexible NFC platform combining a passive circular FPCB tag with a flexible TRF7970A-based reader for temperature and impedance monitoring. The tag integrates an RF430FRL152H transponder, skin-contact impedance electrodes, an analog front end for impedance measurement, and an STS41 temperature sensor. The tag maintained stable data readout within 25 mm, and preserved its response under 1 cm bending. A status-gated, duty-cycled ISO 15693 workflow enabled 0.1 Hz temperature and impedance updates with BLE smartphone visualization.

**Graphical abstract:**

**Figure:**
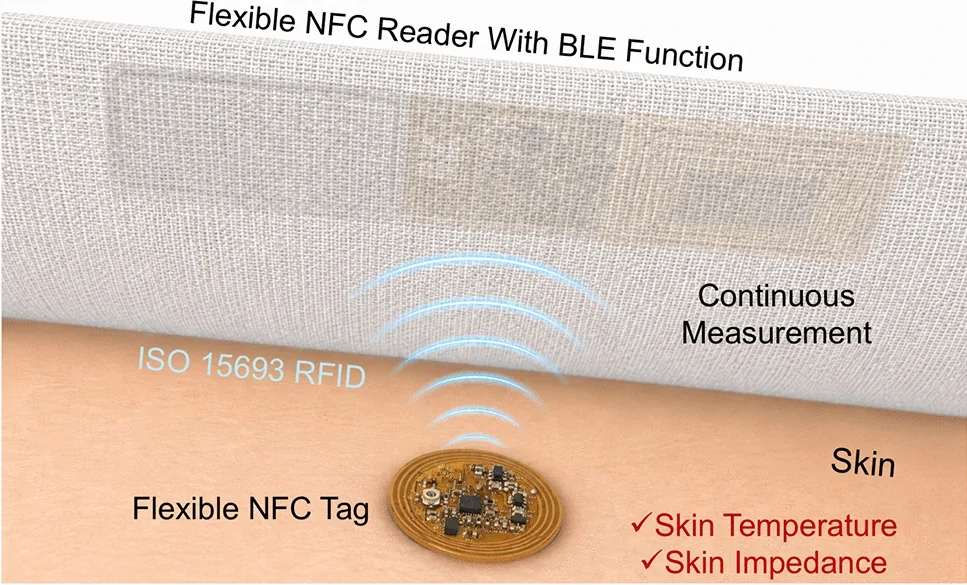

## Introduction

Skin-integrated wearable electronics are increasingly used for continuous physiological monitoring, personalized healthcare, and body-area sensing because they can collect signals directly from the soft, curved, and dynamic surface of the human body.^[[1–4]]^ Compared with conventional rigid electronics, flexible, stretchable, and self-healing platforms can reduce mechanical mismatch at the skin interface and support more stable signal acquisition during natural motion.^[[1–3,5–9]]^ However, long-term wearable monitoring still faces a system-level trade-off: the device must maintain intimate contact with the skin while also integrating power supply, signal conditioning, wireless communication, and data readout. Batteries and active wireless electronics increase the thickness, stiffness, and maintenance burden of skin-mounted devices, which can limit comfort and reliability during prolonged use. This power constraint has motivated broad efforts in energy-autonomous and self-powered systems.^[[10–13]]^

Battery-free wireless sensing provides an attractive route to reduce the burden of skin-mounted electronics by separating the passive skin-interfaced sensor from the power-intensive reader.^[[11,14–22]]^ Previous body-area sensor networks based on stretchable passive tags have shown that skin-mounted tags can be wirelessly coupled to flexible readout circuits placed on textiles, providing an important architecture for distributed physiological sensing without placing rigid active circuits directly on the skin.^[[14]]^ Advances in high-frequency stretchable electronics have further expanded the hardware foundation for soft wireless systems,^[[23]]^ while wireless smart bandage and biointegrated living electronic platforms have demonstrated the importance of combining soft biointerfaces with multimodal physiological sensing.^[[24,25]]^ However, translating these concepts into a practical wearable platform, at current stage, remains challenging. Many existing systems still face challenges in simultaneously provide sufficient wireless power to the tag, maintain reliable sensing under realistic separation and bending conditions, support data acquisition from the passive transponder, and relay the measured signals continuously without a wired connection to the skin-mounted device.

**Figure 1:**
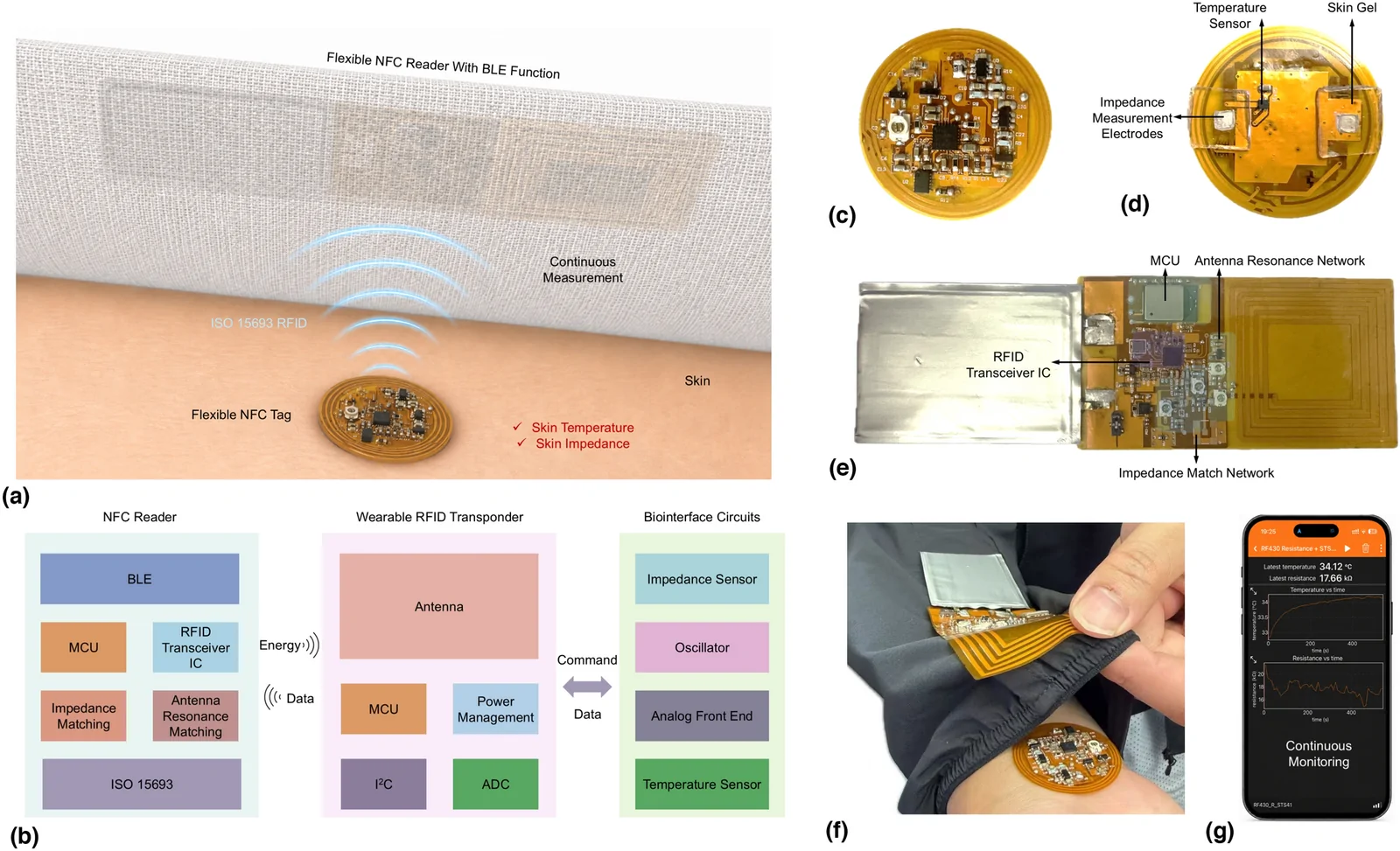
System design of a flexible NFC/RFID wearable platform for continuous skin-interfaced temperature and impedance monitoring. (a) Schematic illustration of our wearable sensing platform (b) System-level block diagram showing the functional partition of the platform. Photographs of the front side (c) and the backside (d) of the circular flexible NFC tag. (e) Photograph of the flexible NFC reader with BLE functionality. (f) Demonstration of the wearable configuration, where the flexible reader is positioned near the textile layer and wirelessly interrogates the skin-mounted flexible NFC tag. (g) Smartphone interface displaying real-time temperature and resistance/impedance readouts transmitted from the reader through BLE, demonstrating the complete sensing-to-display workflow for continuous monitoring.

Here, we report a flexible NFC/RFID wearable sensing platform comprising a battery-free passive circular FPCB sensor tag attached to the skin and a powered flexible reader placed on the textile side. The platform consists of a passive circular flexible printed circuit board (FPCB) sensor tag attached to the skin, and a flexible reader placed on the textile side [Fig. 1(a)]. The passive tag integrates an RF430FRL152H ISO 15693 sensor transponder, a resonant NFC antenna, skin-contact impedance analog circuits and electrodes, and an STS41 temperature sensor. The reader integrates a TRF7970A NFC transceiver, an MCU, RF impedance matching and antenna resonance matching networks, and BLE-based data relay. This system-level partition places the lightweight battery-free tag at the skin interface while moving the complicated and higher-power RF excitation, protocol control, and smartphone communication functions to the external wearable reader.

The overall concept and hardware implementation are summarized in Fig. 1. As illustrated in Fig. 1(a), the flexible reader is positioned near the textile layer and wirelessly interrogates the passive FPCB tag attached to the skin, enabling continuous measurement of skin temperature and skin impedance through ISO 15693 RFID communication. The system-level block diagram in Fig. 1(b) shows a complete energy, command, and data flow: the reader provides RF energy and command signals to the wearable RFID transponder, while the tag transmits the acquired sensing data back to the reader through the same ISO 15693 wireless link. On the tag side, the MCU controls the biointerface circuits for impedance and temperature sensing, including the impedance sensing path, analog front end, oscillator, and temperature sensor. The front and backside photographs of the circular tag show the integrated FPCB layout and the skin-facing sensing interface, including the skin gel region, silver-ink-covered impedance measurement electrodes to prevent electrode oxidation, and the temperature sensor for direct skin-contact measurement [Fig. 1(c) and (d)]. The flexible reader includes the MCU, RFID transceiver IC, impedance matching and antenna resonance networks, and planar antenna required for tag interrogation [Fig. 1(e)]. The wearable demonstration in Fig. 1(f) clearly shows the feasibility and capability of this platform for body-area sensing, where the flexible reader can wirelessly interrogate the skin-mounted passive tag in a wearable configuration. Finally, the smartphone interface in Fig. 1(g) shows that the measured temperature and resistance/impedance data are transmitted to the phone through BLE, demonstrating the platform’s capability for continuous, wireless, skin-interfaced monitoring.

## Methods

The wearable platform consisted of a passive circular FPCB sensor tag and a flexible NFC reader. Both FPCBs were fabricated through PCBWay (Hangzhou, China). The passive tag was designed as the skin-interfaced sensing unit and integrated an RF430FRL152H ISO 15693 sensor transponder (Texas Instruments Inc., USA), a resonant NFC antenna, an STS41 temperature sensor (Sensirion AG, Switzerland), and a custom impedance sensing analog front end. Two commercial skin-contact electrodes were placed on the backside of the tag to define the electrode–skin–electrode impedance path. The electrodes were coated with CI-4040 Ag/AgCl conductive ink (Nagase ChemteX America LLC, USA) to improve electrode stability and reduce oxidation, and a skin gel region was used to improve contact with the skin surface. The flexible reader was implemented on a separate FPCB and integrated a nRF52840 MCU, a TRF7970A NFC transceiver (Texas Instruments Inc.), a source-side impedance matching network, an antenna resonance matching network, a planar reader antenna, and BLE-based data relay. The transmitted temperature and impedance data were received and visualized using the Phyphox smartphone application.

**Figure 2:**
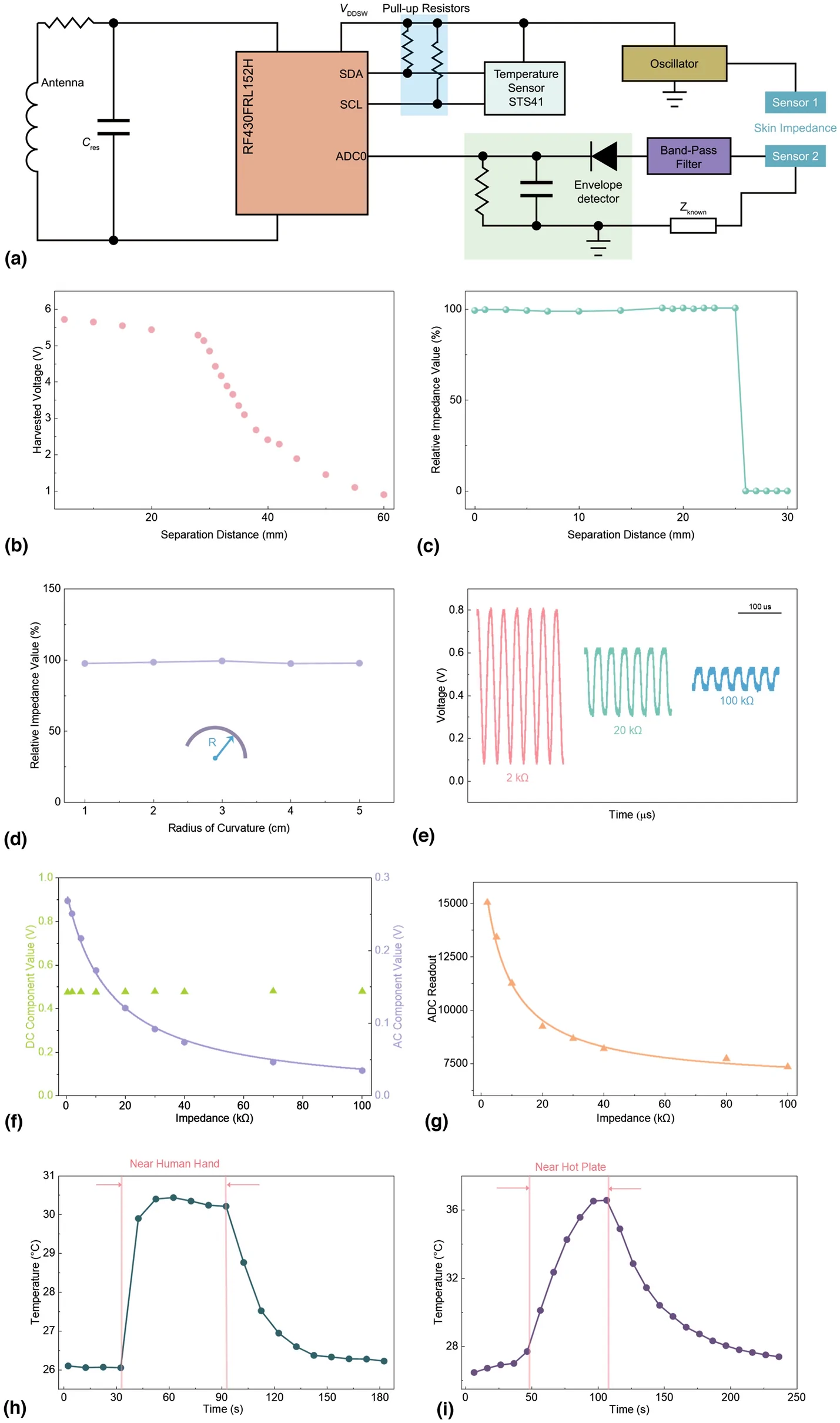
Circuit design and characterization of the passive flexible NFC tag for skin temperature and impedance sensing. (a) Circuit architecture of the passive NFC sensing tag. (b) Harvested voltage of the passive tag as a function of reader-tag separation distance. (c) Relative impedance readout as a function of separation distance. (d) Relative impedance readout under different bending conditions. (e) Representative bandpass filter output waveforms of the impedance sensing circuit measured with reference impedances of 2 kΩ, 20 kΩ, and 100 kΩ. (f) Extracted DC and AC components of the impedance sensing output as a function of input impedance. (g) ADC readout as a function of input impedance. Dynamic temperature response of the passive tag when placed (h) near a human hand, or (i) near a hot plate.

For the distance-dependent energy-harvesting characterization shown in Fig. 2(b), the DC output voltage of the tag-side voltage doubler was measured directly across its positive and negative output terminals using a Keithley DMM6500 digital multimeter. During the measurement, the TRF7970A reader was operated in full power mode, while the passive tag remained in its complete assembled configuration, with all tag-side components and electrical connections, including the RF430FRL152H, STS41 temperature sensor, and impedance sensing analog front end, unchanged and connected. The reader–tag separation was varied while all other circuit and measurement conditions were held constant.

**Figure 3:**
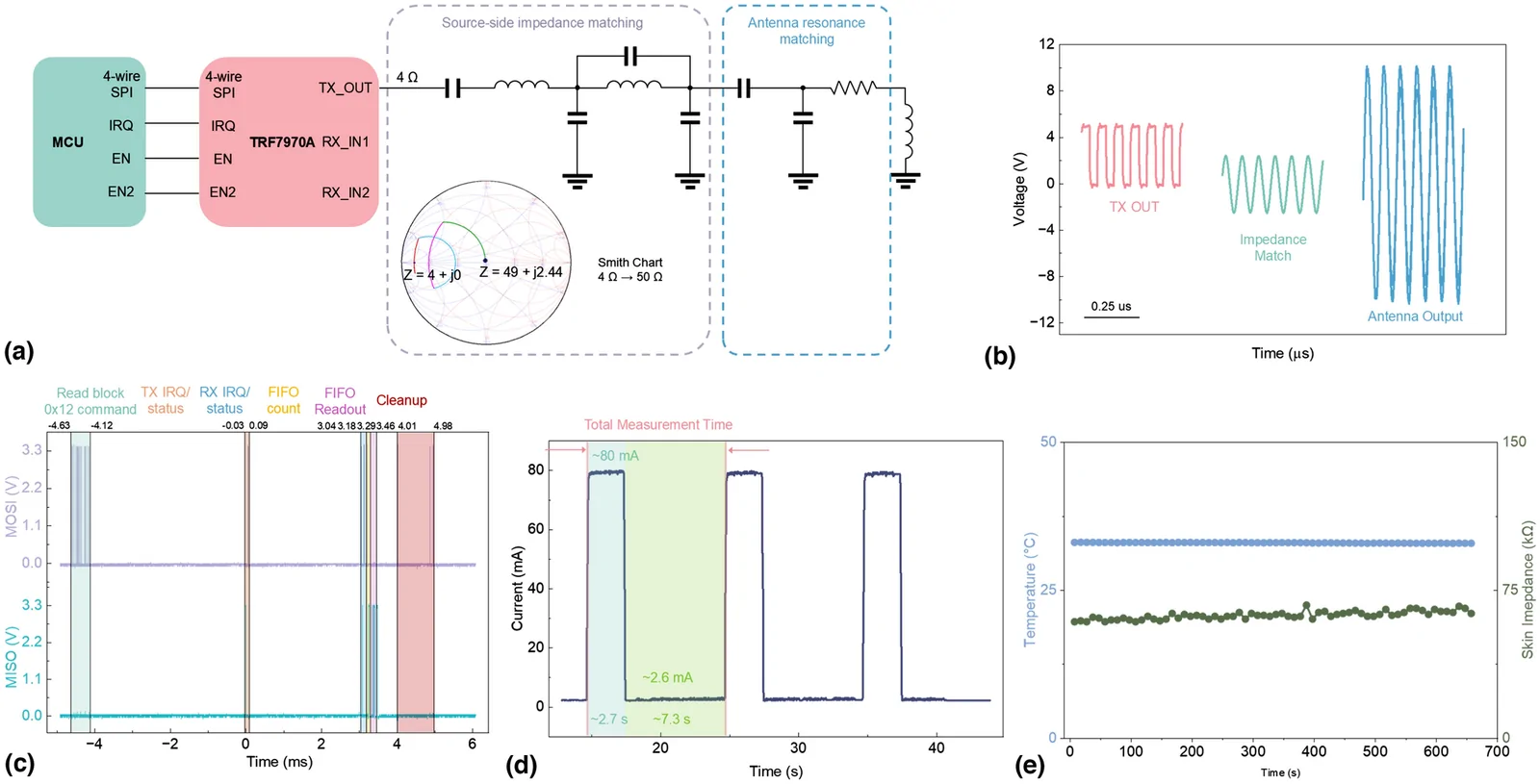
Reader side RF interface, ISO 15693 communication sequence, power consumption, and continuous monitoring demonstration. (a) Circuit diagram of the flexible NFC reader RF front end. The inset Smith chart shows the impedance transformation from the initial low-impedance state to the matched condition. (b) Measured RF waveforms at different stages of the reader output path, including TX_OUT, the impedance-matched node, and the antenna output. (c) Representative ISO 15693 read block transaction used during the status-gated measurement cycle. Block 0 × 12 is shown as the final data-retrieval example after the RF430FRL152H firmware status indicates that sensing data are available. The sequence includes the read block command, TX interrupt/status response, RX interrupt/status response, FIFO count, FIFO readout, and cleanup steps. (d) Current consumption profile of the reader during one measurement cycle. (e) Continuous monitoring result showing simultaneous skin temperature and skin impedance readouts over time.

The reader current-consumption profile shown in Fig. 3(d) was measured using a Keithley DMM6500 digital multimeter operating in current-measurement mode. The multimeter was inserted in series in the reader power supply path between the battery and the reader power switch. Because the measurement was performed upstream of the onboard power-conversion stages, the reported trace represents the total battery-side input current of the complete reader. This measurement includes the onboard 5 V boost converter, 3.3 V low-dropout regulator, nRF52840 MCU, TRF7970A NFC transceiver, and associated peripheral circuitry. The wirelessly powered passive tag and the smartphone were outside this direct electrical current-measurement boundary.

## Results and discussion

### Passive NFC tag design and sensing characteristics

The passive NFC tag was designed as the skin-interfaced sensing unit of the wearable platform. As shown in Fig. 2(a), the RF430FRL152H transponder harvests electromagnetic power from the resonant NFC antenna and supports ISO 15693 communication with the external reader. An STS41 temperature sensor provides digital temperature readout through the I^2^C interface. For impedance sensing, two skin-contact electrodes are attached to the skin to define the electrode–skin–electrode impedance path. A 32.768 kHz oscillator provides an AC excitation signal to this impedance path, and the resulting impedance-dependent signal is first buffered to isolate the electrode interface from the following analog stages. The buffered signal is then passed through a band-pass filter with a 100 Hz–100 kHz passband. The 100 Hz lower cutoff suppresses DC offsets and slow baseline variations from the electrode interface, whereas the 100 kHz upper cutoff attenuates higher-frequency interference while preserving the 32.768 kHz excitation signal with sufficient margin. The filter therefore provides broad band-limiting around the sensing frequency rather than narrow-band frequency selection. After filtering, an envelope detector extracts the peak amplitude of the AC response. This voltage is finally digitized by an internal ADC of the RF430FRL152H. We then evaluated whether this sensing chain could operate under practical wireless and mechanical conditions.

The wireless operating window of the passive tag was first evaluated by varying the separation distance between the reader and tag. The harvested voltage decreased as the reader-tag distance increased, reflecting the expected reduction in near-field coupling strength. The harvested voltage remained above 2 V up to a separation distance of approximately 42 mm, indicating that the near-field link can still provide sufficient tag-side voltage over a centimeter-scale close-range operating distance [Fig. 2(b)]. The 2 V level was used as a practical system-level reference for maintaining adequate voltage headroom at the tag-side voltage-doubler output to support the RF430FRL152H and the connected sensing circuitry. This voltage criterion reflects the availability of harvested energy but does not by itself guarantee successful sensing and wireless data retrieval. Accordingly, the approximately 42 mm distance represents the energy-harvesting range defined by this voltage reference, whereas reliable end-to-end sensing and ISO 15693 communication were maintained within approximately 25 mm. The relative impedance readout remained nearly constant at approximately 99–101% from 0 to 25 mm, confirming normal tag operation and stable impedance data readout within a reader-tag spacing of 2.5 cm [Fig. 2(c)]. Beyond approximately 25 mm, the near-zero values indicate loss of valid sensing and communication and should not be interpreted as a physical impedance value of zero. Because the tag is intended to conform to curved skin surfaces, we further measured the impedance response under different bending radii. The relative impedance values remained almost unchanged for radii of curvature from 1 to 5 cm, indicating that the flexible FPCB structure preserves the impedance sensing response under practical skin-relevant deformation [Fig. 2(d)]. After defining this operating window, we next calibrated the impedance-dependent analog response.

Considering the typical body-surface impedance range,^[[26–30]]^ reference impedances of 2, 20, and 100 kΩ were selected to represent low-, intermediate-, and high-impedance states. The waveforms in Fig. 2(e) were measured at the band-pass-filter output before the envelope detector, where the filtered 32.768 kHz response showed clear impedance-dependent changes in AC amplitude and envelope. This confirms that resistance variation at the skin–electrode interface can be encoded as a measurable AC voltage response for subsequent envelope detection and ADC readout. By extracting the DC and AC components of the output, we found that the AC component exhibited a clear impedance-dependent trend, while the DC component remained comparatively stable [Fig. 2(f)]. This behavior suggests that the impedance information is primarily encoded in the modulated AC response and our band-pass filter functions well, which helps reduce the DC voltage fluctuation due to electrode contact variation, and circuit offset. The ADC readout further established a calibration relationship between the measured signal and the input impedance, enabling the passive tag to convert the analog impedance response into a digital value that can be read wirelessly by the external reader [Fig. 2(g)].

Finally, the temperature-sensing capability was evaluated under two dynamic heating conditions. When the tag was placed near a human hand, the measured temperature increased rapidly during exposure and gradually recovered after removal, demonstrating sensitivity to body-proximal thermal changes [Fig. 2(h)]. A larger thermal transient was observed when the tag was placed near a hot plate, further confirming the dynamic response of the integrated temperature sensor over a broader temperature range [Fig. 2(i)]. These results demonstrate that the passive FPCB tag can support multimodal skin-interfaced sensing by combining impedance-dependent analog readout with digital temperature measurement in a battery-free wireless form factor.

### Reader side RF interface and continuous wearable monitoring

To convert the passive tag response into a complete wearable monitoring system, we therefore developed a flexible reader based on the TRF7970A NFC transceiver, which is controlled by the MCU through a 4-wire SPI interface with IRQ and enable control lines [Fig. 3(a)]. On the RF side, the TRF7970A TX_OUT signal was routed through two cascaded matching stages to efficiently drive the flexible planar reader antenna. Because the TX_OUT port presents a low output impedance of approximately 4 Ω (under full power mode), direct connection to the inductive antenna would lead to poor power transfer and reduced magnetic field strength at the passive tag due to impedance mismatch. A source-side impedance matching network was therefore implemented to transform the low-impedance output toward a 50 Ω RF interface. The Smith chart in Fig. 3(a) shows the impedance transformation from Z = 4 + j0 Ω to Z = 49 + j2.44 Ω, corresponding to a VSWR of 1.057 and a reflection coefficient magnitude of 0.0278. This gives a return loss of approximately 31 dB and a reflected-power fraction below 0.1%, indicating that reflection-related RF loss is negligible at the operating frequency. After this source-side matching stage, the signal was coupled into an antenna resonance matching network to tune the reader antenna at 13.56 MHz and enhance near-field excitation. The source-side network was designed and evaluated in SimNEC at 13.56 MHz. The initial Z = 4 + j0 Ω point represents the nominal TRF7970A TX_OUT source impedance in full power mode used as the simulation input. After the source-side LC matching and harmonic-filter network, the simulated impedance at the R5/J1 50 Ω reference node was Z = 49 + j2.44 Ω. This reference node is located before the separate antenna resonance network. Thus, the inset Smith chart reports the simulated source-side transformation, whereas the waveforms in Fig. 3(b) show the measured RF signals at TX_OUT, the impedance-matched node, and the antenna output after implementation and tuning. The measured waveforms at TX_OUT, after impedance matching, and at the antenna output show the progressive RF signal conditioning through the reader output path [Fig. 3(b)]. In particular, the antenna output waveform confirms that the matching and resonance networks increase the usable RF excitation delivered to the planar antenna [Fig. 3(b)]. This RF front-end design is essential because the passive tag relies entirely on the reader-generated 13.56 MHz magnetic field for both wireless power harvesting and ISO 15693 communication.

To ensure proper communication between reader and tag, the firmware was implemented as a periodic, status-gated measurement loop rather than a direct readout sequence. In each 10 s start-to-start cycle, the reader wakes the TRF7970A, initializes the ISO 15693 interface, turns on the 13.56 MHz RF field, and performs an Inventory command to identify the RF430FRL152H tag. After tag discovery, the reader configures the passive tag using addressed write-block commands. The reader uses standard addressed ISO/IEC 15693 Write Single Block and Read Single Block commands to access the RF430FRL152H FRAM. The functions assigned to blocks 0 × 00, 0 × 01, and 0 × 12 are defined by the RF430FRL152H ROM-firmware memory map rather than by ISO/IEC 15693 itself. In the 4-byte block configuration used here, block 0 × 00 contains the firmware general-control, status, sensor-control, and frequency fields; the reader writes this block to stop or start sampling and reads it to monitor the firmware state. Block 0 × 01 contains the number-of-passes, averaging, interrupt-control, and error-control fields used to configure the acquisition behavior. Block 0 × 12 is the first logging-data block and, for the selected ADC0 and Digital Sensor 1 configuration, contains the 16-bit ADC0 result followed by the 16-bit raw STS41 result. Because the digital temperature conversion requires a finite sampling time, the reader does not immediately read the result mailbox after the start command. Instead, it repeatedly reads block 0 × 00 and monitors the RF430FRL152H firmware status until the state changes from sampling to data available. Only after this confirmation does the reader read block 0 × 12, which serves as the final logging-data block containing the ADC0 result and the raw STS41 temperature data.

Therefore, the read block transaction shown in Fig. 3(c) should be interpreted as a representative ISO 15693 command example within the measurement cycle, with block 0 × 12 shown as the final data-retrieval step. During each transaction, the TRF7970A transmits the command, waits for TX and RX interrupt/status events, checks the FIFO byte count, reads the returned FIFO data, and then clears the communication state before the next command. This status-gated workflow prevents premature reading of incomplete sensor data and explains the current profile in Fig. 3(d). The high-current interval of approximately 80 mA for 2.7 s corresponds to TRF7970A wake-up, RF activation, Inventory, tag configuration, status polling, final data readout, data parsing, and BLE transmission. The reader then enters a low-power interval of approximately 2.6 mA for 7.3 s before the next scheduled measurement, enabling one complete temperature and impedance update every 10 s. Based on the 10 s operating cycle, the cycle-averaged battery-side input current of the complete reader was approximately 23.5 mA. With a nominal reader battery voltage of 3.7 V, this corresponds to an average reader input power of approximately 87 mW.

After each successful block-0 × 12 readout, the MCU parses the returned 4-byte payload, reconstructs the ADC0 code from the first two bytes, and converts it into resistance using the calibration relationship established in Fig. 2(g). The last two bytes are decoded as the raw STS41 temperature output and converted into temperature. The paired resistance and temperature values are then transmitted through BLE to the smartphone interface. The continuous traces in Fig. 3(e) show periodic updates of both skin impedance and temperature, demonstrating that the TRF7970A reader can coordinate RF-powered tag acquisition, status-gated ISO 15693 communication, local data conversion, and BLE readout in a single wearable workflow. The duty-cycled operation of our platform further enables one complete temperature and impedance update every 10 s while keeping the reader in a low-current state between active RFID interrogation windows, supporting low-power long-term wearable monitoring.

The tag-side and reader side results provide complementary validation of the proposed functionally partitioned wearable architecture. The stable impedance readout within 25 mm shows that the passive skin-mounted tag can be interrogated by a nearby textile side reader across a practical close-range spacing, while the preserved response under bending radii of 1–5 cm supports operation on curved body surfaces. On the tag side, integration of the impedance sensing analog front end and digital temperature sensor enables multimodal acquisition without placing a battery or active radio directly on the skin. On the reader side, the RF matching and antenna resonance networks provide the excitation field, the status-gated ISO 15693 workflow prevents retrieval of incomplete measurements, and the MCU and BLE link convert and relay the paired sensor outputs to the smartphone. This division of functions assigns the mechanically lightweight and low-power sensing interface to the skin-mounted tag while moving the more complex and higher-power RF, control, and communication functions to the external reader. The combined results therefore establish the system-level significance of the architecture beyond the validation of its individual components.

## Conclusion

In summary, we developed a flexible NFC wearable platform comprising a battery-free skin-interfaced sensing tag and a powered textile side reader for temperature and impedance monitoring. The circular FPCB tag integrates an RF430FRL152H transponder, skin-contact impedance electrodes, an analog impedance sensing front end, and an STS41 temperature sensor, enabling multimodal sensing without an onboard battery. The passive tag supported stable impedance readout within 25 mm, and preserved its response even under 1 cm bending radius, demonstrating good conformability to the skin surface. The flexible reader provided wireless tag communication, status-gated ISO 15693 data acquisition, local data conversion, and BLE transmission to a smartphone interface. The demonstrated 0.1 Hz operation enabled periodic temperature and impedance updates while keeping the reader in a lower-current state between active RFID windows. These results show the feasibility of separating the lightweight passive skin-mounted tag from the textile side reader, providing a practical route toward low-power, continuous body-area monitoring.

## Data Availability

The data that support the findings of this study are available from the corresponding author upon reasonable request.
